# A scoping review of pre-exposure prophylaxis awareness, acceptability, uptake, adherence, and HIV-related risk behaviors among university students in South Africa

**DOI:** 10.3389/fpubh.2026.1907622

**Published:** 2026-10-15

**Authors:** M. Jacobus Serebane, T. Irene Ramavhoya, T. Edith Mutshatshi, L. Winter Mokhwelepa

**Affiliations:** 1 Department of Nursing Science, Faculty of Health Science, University of Limpopo, Polokwane, South Africa; 2 School of Medicine, Faculty of Health Science, University of Limpopo, Polokwane, South Africa

**Keywords:** HIV prevention, HIV risk behaviors, pre-exposure prophylaxis, PrEP, sexual health, South Africa, undergraduate students, university students

## Abstract

**Background:**

Pre-exposure prophylaxis is an effective biomedical intervention for preventing HIV infection, yet evidence on its awareness, acceptability, uptake and adherence among South African university students remains fragmented.

**Objective:**

To map and synthesize the available evidence on pre-exposure prophylaxis awareness, acceptability, uptake, adherence, and HIV-related risk behaviors among university students in South Africa.

**Methods:**

A scoping review was conducted using Joanna Briggs Institute methodology and reported according to PRISMA-ScR. Studies published between 2014 and 2026 were searched in PubMed/MEDLINE, Scopus, ScienceDirect, CINAHL, African Journals Online and ProQuest Dissertations and Theses/grey literature. Two reviewers independently screened studies and charted data using a predefined extraction tool. Findings were synthesized thematically.

**Results:**

Sixteen studies were included, of which 68.75% (*n* = 11) were quantitative and 31.25% (*n* = 5) qualitative. Evidence was concentrated in KwaZulu-Natal, Gauteng, Limpopo and the Western Cape, with most studies published from 2019 onwards. Six themes emerged: (1) Individual Level: individual perceptions, knowledge and appraisal of HIV and PrEP; (2) Interpersonal Level: interpersonal relationships and social influence on PrEP decision-making; (3) Institutional Level: accessibility, acceptability and responsiveness of PrEP services; (4) Community/social Level: stigma, misconceptions and social norms surrounding PrEP; (5) Structural Level: socioeconomic and health-system conditions shaping PrEP utilization; and (6) HIV Risk Behaviors: patterns of sexual risk and their relationship to PrEP need. Awareness and willingness to use PrEP were generally favorable, but uptake and sustained use were constrained by knowledge gaps, low perceived risk, stigma, side-effect concerns, cost, accessibility and service-related barriers. Risk behaviors included multiple partnerships, inconsistent condom use, substance-associated sexual behavior and socioeconomic pressures.

**Conclusion:**

PrEP utilization is shaped by interacting individual, social, institutional and structural factors. Integrated, youth-friendly services and targeted education addressing HIV risk, stigma and access are needed to strengthen informed PrEP decision-making and prevention.

## Introduction

South Africa continues to experience a substantial Human Immunodeficiency Virus (HIV) burden despite considerable progress in HIV prevention and treatment. According to UNAIDS estimates, approximately 7.7 million people were living with HIV in South Africa in 2023, while an estimated 150,000 new HIV infections occurred during the same year (1). Young people remain an important population in the national HIV response. In 2023, approximately 55,000 new HIV infections occurred among people aged 15–24 years in South Africa (1). The continuing burden of HIV among young people highlights the need for prevention approaches that are accessible, acceptable, and responsive to the circumstances in which young people live, study, and make decisions about their sexual health.

University and college students represent an important population for HIV prevention in South Africa. The transition into higher education is associated with increased independence, changing social and sexual networks, and greater responsibility for individual health decisions. South African studies among university students have reported HIV-related risk behaviours including inconsistent condom use, multiple or concurrent sexual partnerships, and other forms of unprotected sexual activity (2, 3). For example, research among incoming first-year students at a South African university identified patterns of sexual risk behavior over time, demonstrating the continuing relevance of university settings for HIV prevention (2). Similarly, research among undergraduate medical students found gaps in awareness and use of available HIV-prevention measures on campus (3). These findings indicate that higher education institutions represent an important context for understanding both HIV-related risk behaviors and the uptake of HIV prevention interventions.

Pre-exposure prophylaxis (PrEP) provides an additional biomedical option for preventing HIV acquisition among people who are HIV-negative and may be exposed to HIV. South Africa introduced PrEP into its national HIV prevention program in 2016, and PrEP has subsequently become an important component of combination HIV prevention (4, 5). However, the availability of PrEP does not necessarily result in its use. Evidence among South African university students demonstrates gaps in PrEP awareness and knowledge. A study conducted among university students and primary healthcare users in KwaZulu-Natal found that only 12.3% of participants reported having a full understanding of PrEP, with specific knowledge about PrEP remaining low (6). These findings suggest that providing access to PrEP alone may be insufficient if students lack accurate information about its purpose, effectiveness, eligibility, and use.

Importantly, awareness, knowledge, acceptability, willingness, intention, uptake, initiation, adherence, persistence, and discontinuation represent distinct stages or dimensions of the PrEP continuum and should not be used interchangeably. Awareness refers to having heard about PrEP, whereas knowledge concerns understanding what PrEP is, who can use it, and how it should be taken. Acceptability refers to whether PrEP is viewed as acceptable or appropriate, while willingness and intention reflect an individual’s stated readiness or plans to use PrEP. Uptake and initiation refer to the transition from considering PrEP to actually starting it. Adherence concerns taking PrEP according to the recommended regimen, whereas persistence refers to continued use over time; discontinuation refers to stopping PrEP after initiation (7, 8). Evidence from South Africa illustrates these distinctions. Among young people aged 18–24 years, awareness and willingness to use PrEP were considerably higher than actual documented use, demonstrating that interest in PrEP does not necessarily translate into initiation or sustained utilization (7). Similarly, emerging evidence among South African students indicates that knowledge and positive attitudes do not always eliminate concerns or uncertainties surrounding PrEP use (9).

PrEP acceptability and utilization among young people are influenced by individual, interpersonal, social, and structural factors. South African evidence has identified concerns about side effects, uncertainty about PrEP effectiveness, stigma, fear of judgement, partner reactions, misinformation, difficulties accessing services, and the perceived burden of daily pill-taking as factors that may influence PrEP initiation and continued use (7, 10). Recent qualitative research among female college students in Durban found varying levels of PrEP awareness and identified misconceptions, concerns about side effects, HIV-related stigma, fear of partner reactions, and limited access to youth-friendly services as barriers affecting PrEP acceptability and uptake (10). Similarly, research among young people in Gauteng identified barriers operating at individual, interpersonal, institutional, community, and structural levels, including challenges with daily adherence, concerns about disclosure, negative healthcare experiences, limited confidentiality, stigma, and inadequate PrEP information (11). These findings demonstrate that PrEP utilization is a continuum extending beyond initial awareness or uptake and that the higher education and student health environment may shape students’ decisions to initiate, adhere to, persist with, or discontinue PrEP.

Alongside these PrEP-related dimensions, HIV-related risk behaviors constitute an important core concept of this review. University students may experience behaviors and circumstances associated with HIV exposure, including inconsistent condom use, multiple or concurrent sexual partnerships, and unprotected sexual intercourse (2, 3). Such behaviors are relevant to PrEP because PrEP is intended to provide an additional prevention option for individuals who may experience ongoing or changing HIV exposure. However, the presence of HIV-related risk behaviors does not necessarily mean that a student will use PrEP, and PrEP utilization should not be assumed to cause changes in sexual behavior. Rather, mapping both domains allowed the available literature to be examined in terms of what is known about students’ HIV-related risk behaviors and how these behaviors are discussed alongside PrEP awareness, acceptability, uptake, and adherence. Recent research among South African health science students, for example, has examined PrEP knowledge, attitudes, behavioral intention, and concerns about risk compensation, illustrating the importance of considering PrEP-related perceptions alongside HIV-related behavioral factors (9).

Despite the growing availability of PrEP in South Africa, evidence concerning university students remains fragmented across the different components of the PrEP continuum and HIV-related risk behaviors. Existing South African research has examined individual domains such as PrEP awareness and knowledge, willingness to use PrEP, acceptability and barriers to uptake, and barriers to access and adherence among young people (6, 7, 10, 11). Other studies have examined HIV-prevention practices and sexual risk behaviors among university students (2, 3). However, these bodies of evidence are not consistently considered together, and studies vary substantially in the outcomes assessed, terminology used, populations studied, and contexts in which PrEP is delivered.

Recent South African evidence has also tended to focus on particular populations or service-delivery questions rather than specifically on university students. For example, a recent scoping review examined the integration of PrEP with sexual and reproductive health services among adolescent girls and young women in South Africa, highlighting barriers, facilitators, and preferences related to PrEP delivery (12). Another scoping review examined PrEP uptake and utilization among adolescent girls and young women across sub-Saharan Africa, including South African studies, with particular attention to initiation, persistence, and adherence (8). Although these reviews provide important evidence about PrEP among young people and specific priority populations, they do not specifically map the full range of PrEP awareness, acceptability, uptake, and adherence together with HIV-related risk behaviors among university students in South Africa.

Therefore, a South African-specific scoping review is warranted to bring together the fragmented evidence concerning PrEP awareness, acceptability, uptake, adherence, and HIV-related risk behaviors among university students. Mapping these concepts together will provide a clearer understanding of what is known across the PrEP continuum, including where gaps occur between awareness, acceptability, willingness, initiation, uptake, adherence, persistence, and discontinuation, while also characterizing the HIV-related risk behaviors reported within this population. It will further identify the individual, interpersonal, institutional, and structural factors described in the literature and highlight areas where evidence remains limited or inconsistent.

### Study objective

To map and synthesize the available evidence on PrEP awareness, acceptability, uptake, adherence, and HIV-related risk behaviors among university students in South Africa.

### Ethical consideration statement

This scoping review forms part of a master’s dissertation. As this study involves the synthesis of evidence from publicly available literature and does not involve the recruitment or direct involvement of human participants, ethical approval is not required for the conduct of the scoping review. However, the overall master’s research project was reviewed and approved by the Turfloop Research Ethics Committee (TREC). TREC approval number for this study is [REC-0310111-031].

### Conceptual framework

This scoping review adopted the Socio-Ecological Model (SEM) as a guiding conceptual framework to provide a comprehensive understanding of the factors that may shape PrEP awareness, acceptability, uptake, adherence, and HIV-related risk behaviors among university students in South Africa (13). The SEM recognizes that health behaviors are influenced by multiple and interacting levels, extending from individual characteristics to interpersonal relationships, institutional environments, community influences, and broader structural and policy factors. This framework is particularly appropriate for the present review because PrEP-related behaviors and HIV-related risk behaviors among university students are unlikely to be determined by individual factors alone. The framework consists of five domains.

The SEM will therefore provide a structured approach for categorizing and synthesizing determinants across the PrEP continuum, from awareness and acceptability to uptake and adherence, while also mapping HIV-related risk behaviors. The framework will be used to identify patterns, similarities, differences, and gaps in the available evidence across ecological levels. It will not be used to test causal relationships or to assume that a factor identified at a particular level directly causes PrEP use or HIV-related risk behavior. This approach is consistent with the purpose of a scoping review, which is to map the breadth and nature of available evidence and identify areas requiring further investigation. The conceptual framework is also shown in Figure 1 below.

**Figure 1:**
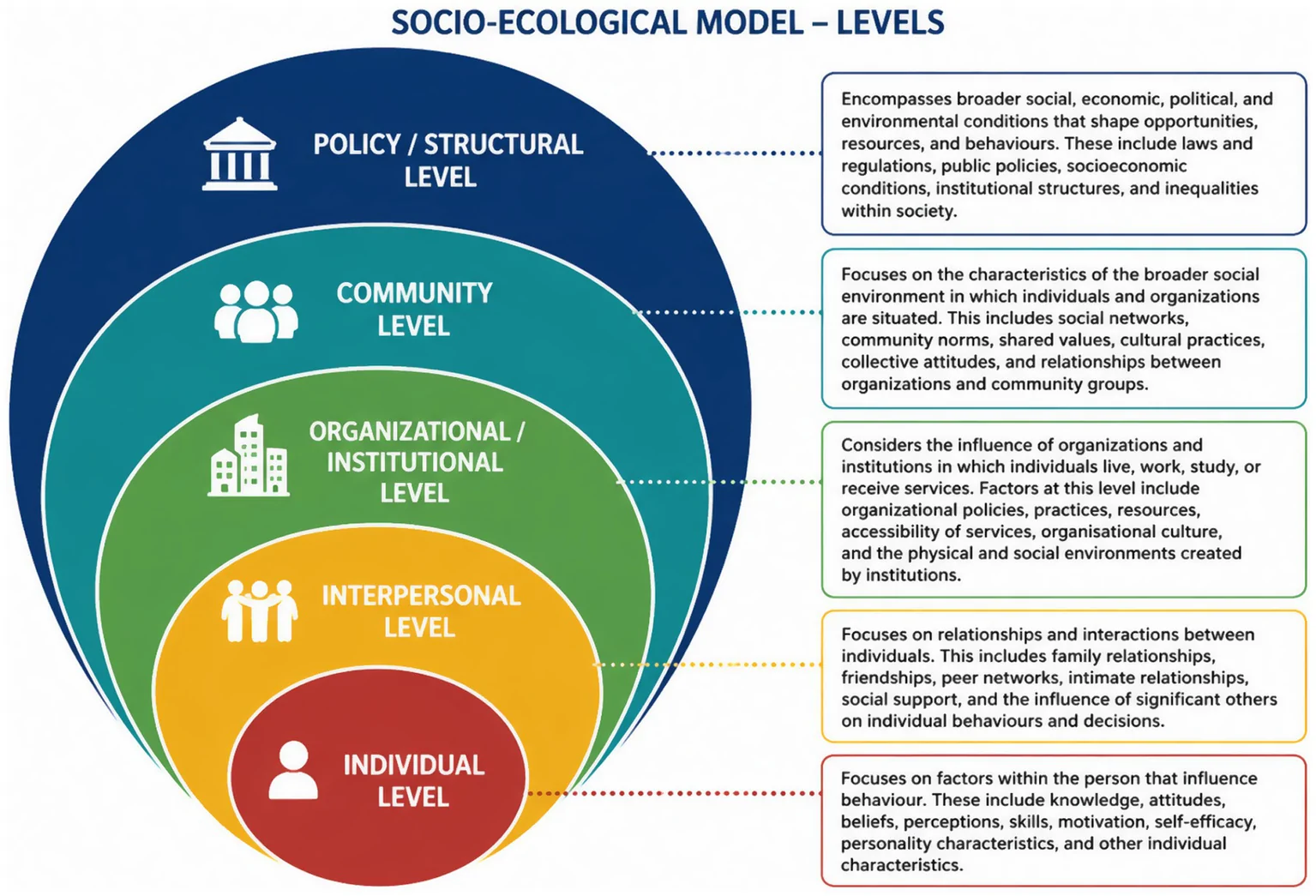
Adopted conceptual framework (13).

## Methods

### Study design

This study adopted a scoping review methodology guided by the Joanna Briggs Institute (JBI) framework for scoping reviews and was reported in accordance with the Preferred Reporting Items for Systematic Reviews and Meta-Analyses extension for Scoping Reviews (PRISMA-ScR) guidelines (14, 15). The JBI methodological framework was used to ensure a systematic and transparent approach to identifying, selecting, and charting relevant literature on factors contributing to the uptake of Pre-Exposure Prophylaxis (PrEP) and associated HIV risk behaviors among undergraduate students in South African universities and colleges. The PRISMA-ScR checklist guided the reporting of the review process to enhance rigor, transparency, and reproducibility.

### Identification of the research question

The review question was structured using the Population, Concept, and Context (PCC) framework:

Population (P): University students enrolled at higher education institutions in South Africa.

Concept (C): PrEP awareness, acceptability, uptake, adherence, and HIV-related risk behaviours.

Context (C): South African higher education institutions and associated student health environments.Therefore, the review question was: What is the available evidence on PrEP awareness, acceptability, uptake, adherence, and HIV-related risk behaviours among university students in South Africa?

## Eligibility criteria

The criteria used to determine study eligibility are presented in Table 1.

**Table 1:** Eligibility criteria.

Eligibility criterion	Inclusion criteria	Exclusion criteria
Population	Undergraduate students enrolled at public or private universities, universities of technology, colleges, or TVET colleges in South Africa. Students of any age were eligible; no age restriction was applied.	Postgraduate students, school learners, out-of-school youth, academic staff, or general populations not enrolled as undergraduate students in South African higher education institutions.
Geographical setting	Studies conducted in South Africa. Multinational studies were included only where South African findings were reported separately or could be extracted separately.	Studies conducted entirely outside South Africa or multinational studies where South African findings could not be separately identified.
HIV status	Studies involving HIV-negative, HIV-positive, mixed, or unspecified HIV-status students were eligible where relevant to the review concepts.	Studies where the population or findings were not relevant to the review concepts.
Review concepts	Studies addressing one or more aspects of the PrEP continuum, including awareness, knowledge, acceptability, willingness, intention, uptake, initiation, utilization, adherence, persistence, or discontinuation, and factors influencing PrEP use. Studies reporting HIV-related risk behaviors, including inconsistent condom use, multiple or concurrent sexual partnerships, transactional sex, and substance use associated with sexual risk, were also eligible.	Studies that did not address PrEP or relevant HIV-related risk behaviors, or studies focusing exclusively on other HIV-prevention interventions without a relevant PrEP component.
Study design	Qualitative, quantitative, and mixed-methods studies, including surveys, cross-sectional studies, cohort studies, interviews, focus groups, and other relevant empirical study designs.	Reviews, systematic reviews, scoping reviews, editorials, commentaries, opinion papers, letters, protocols, and conference abstracts without sufficient empirical findings
Publication type	Peer-reviewed and grey literature, including journal articles, master’s dissertations, and doctoral theses,	Publications without sufficient information to assess eligibility or extract relevant findings.
Language	English-language publications.	Publications not available in English.
Publication period	Studies published between 1 January 2014 and 31 May 2026.	Studies published before 1 January 2014 or after 31 May 2026
Mixed populations	Studies involving undergraduate students alongside other populations were included only when undergraduate-specific results were reported or could be separately extracted.	Studies where undergraduate students were included with other populations, but their findings could not be disaggregated or separately extracted.

### Identification of the relevant studies

A comprehensive literature search was conducted from 1 January 2026 to 31 May 2026 to identify studies addressing PrEP awareness, acceptability, uptake, adherence, and HIV-related risk behaviours among university students in South Africa. The search included the following electronic databases: PubMed/MEDLINE (biomedical, HIV, clinical, and public health literature), Scopus (multidisciplinary health, behavioral, and social science literature), ScienceDirect (health, behavioral, and social science literature), CINAHL (nursing, allied health, and health-service literature), and African Journals Online [AJOL] (African and South African research and regional journals). ProQuest Dissertations and Theses (academic gray literature, including master’s dissertations and doctoral theses) was searched to identify relevant unpublished or non-journal-based research.

The search strategy was developed using keywords and related terms corresponding to the main concepts of the review, namely ‘pre-exposure prophylaxis,” “PrEP,” “HIV prevention,” “university students,” “undergraduate students,” “HIV risk behaviours”, “South Africa”, “HIV prevention”, “sexual health.” Synonyms and variations of the keywords were combined using the Boolean operators AND OR to broaden or narrow the search as appropriate. Truncation and phrase searching were also used where applicable to capture variations of search terms. Database-specific subject headings or controlled vocabulary were incorporated where available. The search was limited to English-language publications published between 1 January 2014 and 31 May 2026. The 12-year period (2014–2026) was selected to capture contemporary and evolving evidence on PrEP while maintaining relevance to current HIV prevention practices. Studies published before 2014 were excluded as they predate the wider implementation and expansion of PrEP and may be less reflective of the current PrEP context. Manual searching was also conducted by screening the reference lists of included studies (backward citation searching) and identifying subsequent studies that cited the included studies (forward citation searching) to capture additional relevant evidence not identified through the electronic database searches. A search strategy is shown below in Table 2.

**Table 2:** Search strategy by database.

Database	Search string	Publication period	Records retrieved
PubMed/MEDLINE	(“Pre-Exposure Prophylaxis”[MeSH Terms] OR “pre-exposure prophylaxis”[Title/Abstract] OR PrEP[Title/Abstract] OR “HIV pre-exposure prophylaxis”[Title/Abstract] OR “tenofovir-based prophylaxis”[Title/Abstract]) AND (“Students”[MeSH Terms] OR “Universities”[MeSH Terms] OR “Education, Higher”[MeSH Terms] OR student[Title/Abstract] OR “university student”[Title/Abstract] OR “college student*”[Title/Abstract] OR undergraduate*[Title/Abstract] OR “tertiary student*”[Title/Abstract] OR “higher education”[Title/Abstract]) AND (“South Africa”[MeSH Terms] OR “South Africa”[Title/Abstract] OR “South African”[Title/Abstract]) AND (“HIV prevention”[Title/Abstract] OR “HIV risk”[Title/Abstract] OR “HIV risk behavio*”[Title/Abstract] OR “HIV exposure”[Title/Abstract] OR “sexual risk behavio*”[Title/Abstract] OR “HIV transmission”[Title/Abstract] OR “sexual behavio*”[Title/Abstract] OR “sexual health”[Title/Abstract] OR condom*[Title/Abstract] OR “multiple sexual partner*”[Title/Abstract] OR “concurrent sexual partner*”[Title/Abstract]))**	01 January 2014–31 May 2026	392
Scopus	TITLE-ABS-KEY(“pre-exposure prophylaxis” OR PrEP OR “HIV pre-exposure prophylaxis” OR “tenofovir-based prophylaxis”) AND TITLE-ABS-KEY(student OR “university student” OR “college student*” OR undergraduate* OR “tertiary student*” OR “higher education”) AND TITLE-ABS-KEY(“South Africa” OR “South African”) AND TITLE-ABS-KEY(“HIV prevention” OR “HIV risk” OR “HIV risk behavio?r*” OR “HIV exposure” OR “sexual risk behavio?r*” OR “HIV transmission” OR “sexual behavio?r*” OR “sexual health” OR condom* OR “multiple sexual partner*” OR “concurrent sexual partner*”)**	01 January 2014–31 May 2026	450
ScienceDirect	(“pre-exposure prophylaxis” OR PrEP OR “HIV pre-exposure prophylaxis” OR “tenofovir-based prophylaxis”) AND (student OR “university student” OR “college student*” OR undergraduate* OR “tertiary student*” OR “higher education”) AND (“South Africa” OR “South African”) AND (“HIV prevention” OR “HIV risk” OR “HIV risk behavio?r*” OR “HIV exposure” OR “sexual risk behavio?r*” OR “HIV transmission” OR “sexual behavio?r*” OR “sexual health” OR condom* OR “multiple sexual partner*” OR “concurrent sexual partner*”)**	01 January 2014–31 May 2026	351
CINAHL	(MH “Pre-Exposure Prophylaxis+” OR “pre-exposure prophylaxis” OR PrEP OR “HIV pre-exposure prophylaxis”) AND (MH “Students, University+” OR MH Students+ OR student OR “university student” OR “college student*” OR undergraduate* OR “tertiary student*” OR “higher education”) AND (“South Africa” OR “South African”) AND (MH “HIV Infections+” OR “HIV prevention” OR “HIV risk” OR “HIV risk behavio?r*” OR “HIV exposure” OR “sexual risk behavio?r*” OR “HIV transmission” OR “sexual behavio?r*” OR “sexual health” OR condom* OR “multiple sexual partner*” OR “concurrent sexual partner*”)**	01 January 2014–31 May 2026	300
African Journals Online (AJOL)	(“pre-exposure prophylaxis” OR PrEP OR “HIV pre-exposure prophylaxis” OR “HIV prevention”) AND (student OR “university student” OR “college student*” OR undergraduate* OR “tertiary student*” OR “higher education”) AND (“South Africa” OR “South African”) AND (“HIV risk” OR “HIV risk behavio?r*” OR “HIV exposure” OR “sexual risk behavio?r*” OR “HIV transmission” OR “sexual behavio?r*” OR “sexual health” OR condom* OR “multiple sexual partner*” OR “concurrent sexual partner*”)**	01 January 2014–31 May 2026	05
ProQuest Dissertations and Theses / OpenGrey	(“pre-exposure prophylaxis” OR PrEP OR “HIV pre-exposure prophylaxis” OR “tenofovir-based prophylaxis” OR “HIV prevention”) AND (student OR “university student” OR “college student*” OR undergraduate* OR “tertiary student*” OR “higher education”) AND (“South Africa” OR “South African”) AND (“HIV risk” OR “HIV risk behavio?r*” OR “HIV exposure” OR “sexual risk behavio?r*” OR “HIV transmission” OR “sexual behavio?r*” OR “sexual health” OR condom* OR “multiple sexual partner*” OR “concurrent sexual partner*”)**	01 January 2014–31 May 2026	02

The review focused on empirical research, including qualitative, quantitative, and mixed-methods studies, to capture both numerical evidence and in-depth perspectives on PrEP awareness, acceptability, uptake, adherence, and HIV-related risk behaviors among undergraduate students in South Africa. Eligible publication types included peer-reviewed journal articles as well as relevant master’s dissertations and doctoral theses identified through the grey-literature search. Grey literature helped the authors to identify relevant evidence that may not have been captured in the peer-reviewed literature, particularly research conducted within South African higher education settings. Conference abstracts without sufficient methodological and empirical information were excluded. Grey-literature records underwent the same screening and eligibility assessment procedures as peer-reviewed publications.

### Selection of relevant studies

The selection of relevant studies was conducted using a structured, systematic, and reproducible screening process based on the predefined eligibility criteria (see Supplementary material 1). A total of 1,500 records were retrieved from the electronic databases and other information sources. All retrieved records were imported into Zotero (version 8) for reference management and organization. Duplicate records were identified and removed prior to screening, with the number of duplicates removed documented as part of the study selection process.

Following deduplication, the remaining records were independently screened by two reviewers (M.J.S. and L.W.M.) at the title and abstract level against the predefined inclusion and exclusion criteria. Each reviewer independently recorded an inclusion or exclusion decision for every record, and records considered potentially relevant by either reviewer were retained for full-text assessment. No formal pilot screening exercise was conducted before commencement of the main screening; however, the reviewers applied the eligibility criteria consistently throughout the screening process. Full-text articles were subsequently retrieved for all records considered potentially eligible and were independently assessed by the same two reviewers (M.J.S. and L.W.M.). At the full-text stage, each reviewer independently assessed the article against all predefined eligibility criteria and documented the reason for exclusion for studies that did not meet the criteria. This ensured that decisions at the full-text stage were traceable and could be verified during the review process.

Disagreements at either the title/abstract or full-text screening stage were initially discussed between the two reviewers and resolved through consensus. Where consensus could not be achieved, a third reviewer (T.I.R.), an experienced senior member of the research team, was consulted and made the final eligibility decision. In addition, the senior research team members reviewed the final set of included studies to identify any remaining inconsistencies or uncertainties in the application of the eligibility criteria. Any issues identified during this review were discussed collaboratively and resolved with reference to the predefined eligibility criteria rather than through *post hoc* modification of the criteria.

The screening process was documented throughout, including the number of records identified, duplicates removed, records screened, full-text articles assessed, full-text articles excluded and the corresponding reasons for exclusion, and studies ultimately included in the review. These data were used to construct the PRISMA-ScR flow diagram (Figure 2), thereby providing a transparent account of the study selection process. Following completion of title and abstract screening, full-text assessment, and resolution of disagreements, 16 studies met all predefined eligibility criteria and were included in the final scoping review.

**Figure 2:**
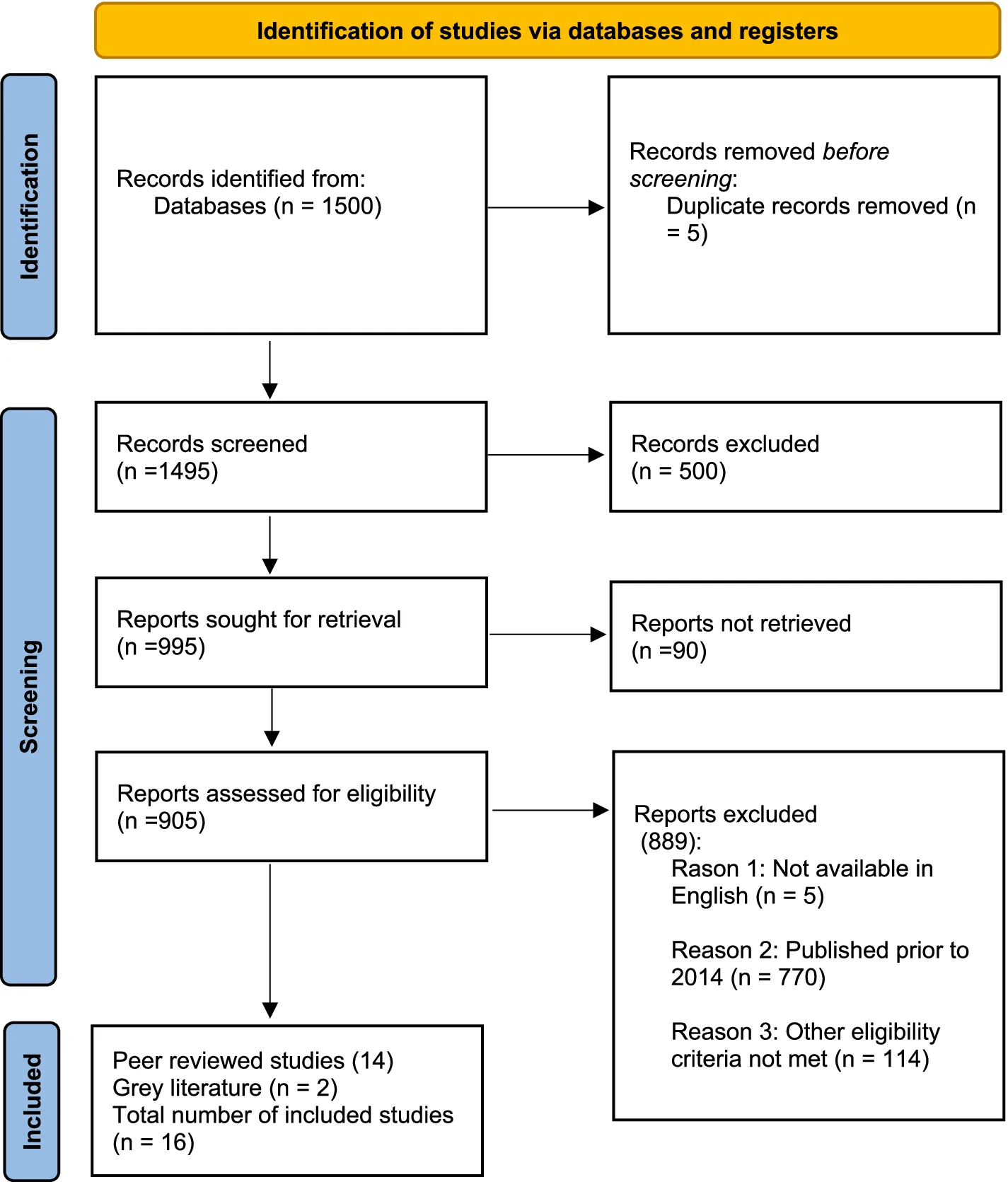
A Prisma flow diagram representing the study selection (15).

### Quality appraisal

Methodological quality appraisal was not undertaken in this scoping review, as critical appraisal is not a mandatory requirement for scoping reviews and is dependent on the purpose and methodological framework adopted. Given that the primary objective of this review was to systematically map the extent, range, and characteristics of the available evidence on PrEP awareness, acceptability, uptake, adherence, and HIV-related risk behaviors among undergraduate students in South Africa, rather than to assess the effectiveness of interventions or determine the certainty of evidence, formal methodological appraisal was considered unnecessary. Consequently, study inclusion was based on the predefined eligibility criteria, and no study was excluded on the basis of methodological quality or risk of bias.

### Data extraction

A data extraction tool was developed using Microsoft Excel to systematically capture relevant information from the included studies (see Supplementary material 2). The key elements included in the extraction tool were study authors, year of publication, study design, and main findings relevant to the review question. Zotero (version 8) was used as the reference management software to organize and manage the retrieved literature and references throughout the review process. Data extraction was conducted independently by two reviewers (M.J.S. and L.W.M.) to enhance accuracy and reduce the risk of bias. Any discrepancies identified during the extraction process were resolved through discussion and consensus. Where disagreements persisted, a third reviewer (T.I.R.) was consulted to ensure agreement on the final extracted data. The extracted data were subsequently used to support the synthesis of findings in the review. Summary characteristics of the included studies are presented in Table 3.

**Table 3:** Summary characteristics of the included studies.

Author and year	Study method/ Design	Data collection method	Study province	Setting (Institution)	Sample size (N)	Age range	Gender	Prep outcome	HIV risk behavior	Relevant barriers	Principle findings
Haffejee et al., 2023 (6)	Quantitative study	Self-administered questionnaire	Kwa-Zulu Natal	Durban University of Technology	282	18–24	Both Males and Females	Low knowledge and awareness of PrEP and its availability	High-risk groups had limited understanding of PrEP use; specific behaviors	Limited information and poor awareness of PrEP services	Knowledge and awareness of PrEP among high-risk groups in KwaZulu-Natal were generally low, with very limited understanding of its use and availability at local clinics. Slightly better awareness was observed among university students and individuals with higher education levels, but overall knowledge remained insufficient.
Ntho et al., 2026 (9)	Quantitative study	Self-administered, structured online questionnaire via Google Forms	Capricorn District,	University of Limpopo	350	18 and above	Both Males and Females	Good PrEP knowledge attitude and behavioral intention towards HIV PrEP.	HIV risk behavior was not directly measured. However, 48.6% believed PrEP could promote risky sexual behavior; 9.9% agreed that multiple sexual partners are acceptable while using PrEP; and 6.3% agreed that condoms are unnecessary when taking PrEP.	Knowledge gaps concerning PrEP eligibility and dosing; limited access to information; concerns about cost; accessibility and prescription requirements; misconceptions about risk compensation; broader concerns about rural access and youth-friendly HIV services.	Health science students showed generally strong PrEP knowledge, positive attitudes, and high willingness to use PrEP. Older students and those in later years of study performed better. However, misconceptions about risk compensation and practical barriers involving information, cost, and access may limit actual PrEP uptake. The authors recommend integrating PrEP education into curricula and strengthening campus-based HIV prevention services.
Potsane et al., 2025 (17)	Quantitative study	Self-administered questionnaire	Gauteng	Six selected universities (Unspecified)	537	18 and above	Both Males and Females	High awareness but low PrEP uptake, particularly among students at increased HIV risk	HIV risk was associated with low PrEP uptake; specific behaviors NR	Limited knowledge, social and situational factors, and low perceived benefits	University students in Gauteng showed high awareness of PrEP but low uptake, especially among those at risk of HIV infection. Uptake was influenced by knowledge, social and situational factors, and perceived benefits, highlighting the need for targeted, youth-friendly interventions.
Manyaka, 2024 (18)	Quantitative study	Self-administered questionnaire	Western Cape	University of the Western Cape	51	18–24	Both Males and Females	High acceptance of CAB-LA injectable PrEP	Specific risk behaviors NR	Fear of injections, side effects, cost, and frequent clinic visits	University students showed high acceptance of CAB-LA injectable PrEP due to its effectiveness and privacy advantages, but concerns about injections, side effects, costs, and clinic visits reduced uptake. The study highlights the need for improved HIV prevention education and accessible, affordable services to support implementation.
Ndabarora and Mchunu (2014) (19)	Quantitative study	Self-administered questionnaire	KwaZulu-Natal	University of KwaZulu-Natal campus	335	17–48	Both Males and Females	PrEP not specifically assessed; awareness and use of HIV prevention services assessed	Condom use and VCT were the most commonly used prevention practices; peer and wellness services were underused	Stigma, confidentiality concerns, and poor awareness of available services	Awareness of HIV prevention services was the main driver of utilization among University of KwaZulu-Natal students, with condoms and VCT being most commonly used while peer and wellness services were underused. Despite high knowledge, uptake was limited by stigma, confidentiality concerns, and poor awareness, highlighting the need to strengthen and expand campus-based interventions.
Musakwa et al., 2021 (20)	Quantitative study	Self-administered questionnaire	Gauteng	3 selected Universities in Johannesburg (Unspecified)	792 University of Johannesburg students	18–25	Both Males and Females	PrEP not assessed	HIV testing uptake was limited; other risk behaviors NR	Long waiting times, service costs, poor health literacy, academic conflicts, negative staff attitudes, and inadequate health information	HIV testing and TB screening uptake among first-year students in Johannesburg was limited by barriers such as long waiting times, costs, poor health literacy, and academic schedule conflicts. Despite campus clinics, students often used external services due to negative staff attitudes and insufficient university health information.
Shamu, et al., 2025 (21)	Qualitative study	Semi-structured interviews	Gauteng	City of Tshwane	22	18–24	Females	Findings informed delivery of long-acting PrEP; uptake was influenced by contraceptive experiences	Sexual and contraceptive risk behaviors were not directly specified	Fear of side effects, stigma, negative social influences, and health-system limitations	Contraceptive uptake among female university students in South Africa is influenced by individual, social, community, and health system factors, including perceived benefits and supportive relationships. However, fear of side effects, stigma, and negative influences hinder use, suggesting the need for youth-friendly services, trained providers, and improved HIV prevention education.
Haffajee, et al., 2022 (22)	Qualitative study	Semi-structured interviews	Kwazulu-Natal	University of Kwazulu-Natal	12	18–24	Both Males and Females	Students initiated PrEP because of perceived HIV risk and valued its protective benefits; adherence was challenging	Multiple sexual partners and inconsistent condom use	Forgetfulness, side effects, service disruptions, misconceptions that PrEP replaces condoms	University students in KwaZulu-Natal initiated PrEP mainly due to perceived HIV risk from multiple partners and inconsistent condom use, valuing its protective benefits. However, adherence was challenged by forgetfulness, side effects, service disruptions, and misconceptions that PrEP can replace condoms, highlighting the need for ongoing support and integrated prevention strategies.
Pillay, et al., 2025 (25)	Quantitative study	Self-administered questionnaire	KwaZulu-Natal	University of Kwazulu-Natal	500	Above 18	Both Males and Females	Not Reported	Inconsistent condom use, inadequate knowledge of anal-sex risks, and other unsafe sexual behaviors	Misconceptions and inadequate HIV-prevention education	Inadequate HIV knowledge among KwaZulu-Natal University students is linked to higher HIV risk and unsafe sexual behaviors, including inconsistent condom use and low awareness of anal sex risks. The study highlights the need for comprehensive, youth-focused HIV education to correct misconceptions and improve understanding of prevention methods like PrEP and PEP.
Shamu, et al., 2024 (26)	Qualitative study	Semi-structured interviews	Gauteng	City of Tshwane	22 university students	18–24	Females	Participants preferred CAB-LA injection over oral PrEP.	HIV risk behaviors not directly reported	Preference was influenced by convenience, discretion, and lifestyle fit; other barriers NR	Young South African women preferred CAB-LA injectable PrEP over daily oral PrEP and the dapivirine vaginal ring due to its convenience, discretion, and better fit with their lifestyles.
Shamu, et al., 2025 (23)	Quantitative study	Self-administered questionnaire	Gauteng	City of Tshwane	400	18–24	Females	Participants reported good awareness and acceptance of PrEP.	HIV risk behaviors not directly reported	Cost, accessibility, provider attitudes, and service location	The study identified that female students in South African tertiary institutions prefer accessible and affordable long-acting injectable Pre-Exposure Prophylaxis (PrEP) services offered by supportive healthcare professionals at campus clinics over public ones.
Pillay, et al., 2026 (27)	Quantitative study	Self-administered questionnaire	KwaZulu-Natal	Durban University Technology	500	Above 18	Both Males and Females	Not Reported	Risky sexual behavior, multiple partners, alcohol and drug use before sex, and HIV seropositivity	Substance use and associated sexual decision-making	The study found a strong link between risky sexual behaviors, substance use, and HIV among South African university students. Factors such as heavy alcohol and drug use before sex increased the likelihood of multiple partners, particularly among males.
Pillay, et al., 2023 (28)	Quantitative study	Self-administered questionnaire	KwaZulu-Natal	University of KwaZulu-Natal	500	Above 18	Both Males and Females	Not Reported	Risky sexual behavior and HIV seropositivity	Socioeconomic conditions; specific barriers NR	The study reported that socioeconomic factors play a crucial role in influencing risky sexual behaviors and HIV seropositivity among university students in KwaZulu-Natal, South Africa
Tabata N. P, 2018 (29)	Qualitative study	Semi-structured interviews	Western Cape	Cape peninsula University	31	18–26	Both Males and Females	Not Reported	Students are still afraid of being seen entering campus health clinics, Inconsistent condom use, multiple partners, and substance use	Socioeconomic challenges, poor family communication, limited HIV knowledge, stigma, misconceptions, and negative perceptions of health services	University students in the Western Cape engage in risky sexual behaviors such as inconsistent condom use, multiple partners, and substance use, driven by socioeconomic challenges and poor family communication. Limited HIV knowledge, misconceptions, and negative perceptions of health services due to stigma highlight the need for comprehensive education, youth-friendly services, and stronger peer support systems.
Murudi-Manganye et al., 2020 (30)	Quantitative study	Self-administered questionnaire	Limpopo	Vhembe Technical Vocational Education and Training (TVET) college	330	21–24	Both Males and Females	Not Reported	High prevalence of risky sexual behavior	Demographic, environmental, and socioeconomic factors	The study found a high prevalence of risky sexual behaviors among students, driven by an interplay of demographic, environmental, and socioeconomic factors.
Mthembu et al., 2019 (24)	Qualitative study	Semi-structured interviews	KwaZulu- Natal	Durban university of Technology	20	18–24	Both Males and Females	Not Reported	Inconsistent condom use, unprotected sex, and multiple partners	Alcohol use, peer pressure, masculinity norms, transactional sex, relationship-based trust, poor perceptions of free condoms, lack of preparedness at sexual debut, and socioeconomic pressure	The study found that declining condom use among South African university students is driven by alcohol use, peer pressure, masculinity norms, and transactional sex, leading to unprotected sex and multiple partners. Inconsistent condom use was also linked to trust in relationships, poor perceptions of free condoms, lack of preparedness at sexual debut, and socioeconomic pressures.

### Collation, summary, and reporting of results

The collation, summary, and reporting of results followed a structured thematic synthesis approach (16). Extracted data from the included studies were systematically organized and analyzed to identify recurring patterns, similarities, and differences across the literature. An initial coding process was conducted whereby meaningful text segments related to PrEP uptake and HIV risk behaviors were assigned descriptive codes. These codes were then compared and grouped based on conceptual similarity.

Through an iterative process, related codes were clustered to form broader categories, which were subsequently refined into overarching themes. This thematic synthesis allowed for the integration of both qualitative and quantitative findings, enabling a comprehensive mapping of factors influencing PrEP uptake and associated HIV risk behaviors among undergraduate students. The developed themes were reviewed and refined to ensure they accurately reflected the extracted data and aligned with the objectives of the review. The final synthesis was presented in a narrative format supported by tables and descriptive summaries to provide a clear and structured representation of the evidence. Summary of the developed themes is shown in Table 4 below.

**Table 4:** Summary of the developed themes.

Level	Proposed overarching theme	Definition	Key determinants	Supporting studies	Main interpretation
1. Individual	Individual perceptions, knowledge and appraisal of HIV and PrEP	Individual knowledge, beliefs and perceptions that shape how students understand HIV risk, evaluate PrEP and assess their ability to use it.	HIV and PrEP knowledge; awareness of prevention services; perceived HIV risk; perceived benefits; perceived barriers; self-efficacy; motivation; adherence capacity	Potsane et al. (17), Ndabarora and Mchunu (19), Haffejee et al. (6), Haffajee et al. (22), Pillay et al. (25), Mthembu et al. (24), Murudi-Manganye et al. (30), Manyaka (18), Shamu et al. (26), and Musakwa et al. (20)	Individual knowledge, perceptions and motivation influence PrEP consideration, initiation and continued use, but do not operate independently of social and environmental conditions.
2. Interpersonal	Interpersonal relationships and social influence on PrEP decision-making	The influence of partners, peers and close social relationships on students’ perceptions, negotiations and decisions regarding PrEP use.	Partner influence; partner negotiation; peer influence; peer pressure; social support; relationship trust; relationship status; perceptions of PrEP users; gender and masculinity norms	Shamu et al. (21, 23), Tabata (29), Mthembu et al. (24), and Pillay et al. (25)	PrEP decision-making is socially embedded. Supportive partners and peers can facilitate uptake, whereas relationship pressure, mistrust and gender norms can discourage utilization.
3. Institutional	Accessibility, acceptability and responsiveness of PrEP services	The extent to which institutions provide accessible, confidential, acceptable and responsive PrEP services that enable students to initiate and continue utilization.	PrEP availability; campus clinic accessibility; confidentiality; provider attitudes; opening hours; waiting times; cost; student-friendly services; medication availability; service continuity	Musakwa et al. (20), Shamu et al. (21, 23), Shamu et al. (26), and Pillay et al. (28)	Positive individual motivation may not result in utilization when institutional services are inaccessible, unaffordable, unavailable or perceived as judgmental or inconvenient.
4. Community/social	Stigma, misconceptions and social norms surrounding PrEP	The wider social meanings, attitudes and norms that influence whether PrEP is viewed as acceptable, necessary, shameful or associated with negative judgement.	HIV-related stigma; PrEP-related stigma; fear of judgement; misconceptions; social norms; perceptions of PrEP users; community support; positive prevention norms; masculinity expectations	Shamu et al. (21, 23), Tabata (29), Mthembu et al. (24), Pillay et al. (25), and Manyaka (18)	Community meanings attached to PrEP can either normalize prevention or frame PrEP use negatively, limiting disclosure, service seeking and sustained utilization.
5. Structural	Socioeconomic and health-system conditions shaping PrEP utilization	The broader socioeconomic, policy and health-system conditions that influence whether students have the resources and opportunities to access and sustain PrEP use.	Socioeconomic circumstances; affordability; health-policy environment; medication supply; health-system capacity; gender inequality; broader implementation barriers; continuity of care	Musakwa et al. (20), Shamu et al. (21, 23), Shamu et al. (26), and Pillay et al. (28)	Structural conditions shape whether students can act on their intentions. Resource constraints, inequality and health-system limitations may restrict access even when PrEP is viewed positively.
Outcome	PrEP utilization and HIV risk reduction	The behavioral pathway through which awareness and intention may lead to PrEP initiation, adherence, continued utilization and potential reduction in HIV risk.	PrEP awareness; intention; initiation; adherence; continued utilization; continuity of care; HIV-risk reduction	Outcome pathway synthesized across the included studies	PrEP utilization represents the outcome of interacting individual, interpersonal, institutional, community and structural conditions.

## Results

This study included 16 relevant studies. Six themes emerged from this study.

The findings indicate a clear increase in research activity on PrEP among South African university and college students in recent years. Half of the included studies (50%, *n* = 8) were published during 2024–2026, making this the most represented publication period, while studies published between 2019 and 2023 accounted for 37.5% (*n* = 6) of the evidence base. In contrast, only 12.5% (*n* = 2) of the studies were published between 2014 and 2018. This study identifies 2025 as the peak publication year, with five studies (33.3%) published during this year. This increasing concentration of publications in the most recent period suggests that PrEP among the university and college students has become a progressively more prominent research priority, potentially reflecting the growing recognition of university and college students as an important population within HIV prevention. However, the relatively small number of studies published during the earlier period also indicates that the evidence base remains comparatively recent, with limited longitudinal accumulation of evidence from which to assess how PrEP awareness, uptake, adherence and decision-making have evolved over time.

Regarding study design, quantitative research predominated, accounting for 68.75% (*n* = 11) of the included studies, while qualitative studies represented 31.25% (*n* = 5) of the evidence base. This predominance indicates that the current evidence on PrEP among South African university and college students is largely derived from quantitative, cross-sectional approaches. Quantitative studies are valuable for mapping the extent of PrEP awareness, acceptability, perceived risk, uptake and associated factors, as they provide estimates of prevalence and identify statistical associations between PrEP-related outcomes and individual or contextual characteristics. However, the predominance of cross-sectional designs also limits the depth of interpretation that can be drawn from the evidence. Such studies provide a snapshot of participants’ experiences at a particular point in time and can identify relationships between variables, but they cannot adequately establish temporal changes, causal relationships or explain how and why students make decisions regarding PrEP initiation, adherence and continued utilization.

The geographical distribution demonstrates a marked concentration of research in KwaZulu-Natal, Gauteng, Limpopo and the Western Cape, with comparatively limited representation from other South African provinces. This concentration suggests that the available evidence does not provide an even geographical representation of university and college students nationally. In particular, the absence or limited representation of studies from several provinces indicates that students in these settings remain less visible within the current evidence base. This is important because experiences of PrEP access, HIV risk, sexual health services and prevention needs may differ according to local socioeconomic circumstances, institutional resources, healthcare accessibility and the characteristics of the surrounding communities. The geographical concentration should therefore be considered when interpreting the transferability of the findings, as evidence generated predominantly in a small number of provinces may not fully capture the experiences of students attending institutions in less-researched settings.

The observed geographical pattern may partly reflect the distribution of established higher-education institutions, research capacity and HIV-related research networks within the represented provinces. However, the concentration cannot be assumed to correspond directly with provincial HIV burden, because the geographical distribution of the included studies reflects where research was conducted rather than the distribution of HIV epidemiological burden itself. The Geographical locations of the included studies are shown in Figure 3 below.

**Figure 3:**
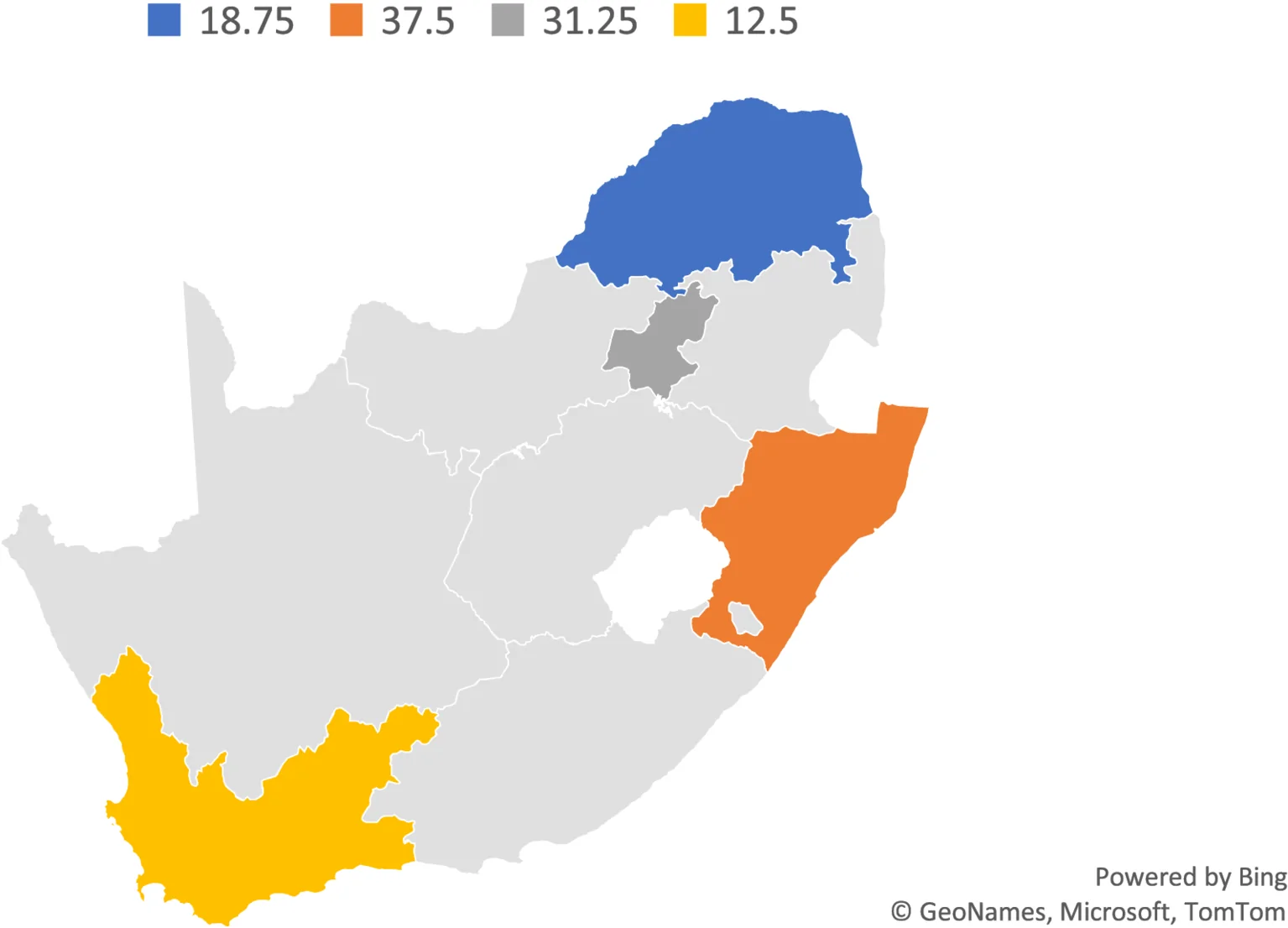
A map showing the geographical locations of the included studies.

Participants in the majority of the research that made up this review were between the ages of 18 and 24. Many young adults participating in postsecondary education, especially undergraduate university and college programs, are between the ages of 18 and 24. Since many students are sexually active during this time and may thus benefit from HIV preventive therapies like PrEP, this age group is also pertinent to HIV prevention research.

## Theme 1

### Individual level: individual perceptions, knowledge and appraisal of HIV and PrEP

The evidence indicates that individual-level perceptions and knowledge are important entry points for PrEP utilization, but they do not, on their own, determine whether students initiate or sustain PrEP. Across the included studies, knowledge and awareness ranged from substantial gaps to relatively high levels, suggesting that students’ understanding of PrEP is not uniform across South African higher-education settings. Ntho et al. (9) reported high PrEP knowledge, positive attitudes and strong behavioral intention among health sciences students, while Potsane et al. (17) similarly identified high awareness but low PrEP uptake. In contrast, Haffejee et al. (6) found inadequate knowledge and awareness of PrEP among high-risk groups, indicating that awareness remains uneven despite increasing availability of PrEP information (6). Manyaka (18) further demonstrated that students may express high acceptance of CAB-LA because of its effectiveness and privacy advantages, yet concerns about injections, side effects, cost and clinic attendance can undermine actual utilization.

The most important pattern across these findings is therefore the disconnect between awareness, intention and utilization. Positive knowledge and attitudes appear to create the conditions for PrEP consideration, but they do not necessarily translate into behavior. This is particularly evident in the contrast between Ntho et al. (9), where 80.3% of students reported high behavioral intention to use PrEP, and Potsane et al. (17), where awareness was high but uptake remained low. The findings suggest that students make an individual appraisal of PrEP in which perceived HIV vulnerability and preventive benefits are weighed against concerns about side effects, cost, convenience and the perceived necessity of PrEP. Studies reinforced this interpretation: students-initiated PrEP because they perceived themselves to be at risk and valued its protective benefits, but maintaining use was subsequently challenged by forgetfulness, side effects, service disruptions and misconceptions about replacing condoms (19–22).

Thus, the evidence suggests that knowledge is necessary but insufficient. The individual-level barrier is not simply a lack of information; rather, it concerns whether students perceive PrEP as personally necessary, acceptable and manageable within their everyday lives. Musakwa et al. (20) similarly showed that awareness of health services does not automatically result in utilization when students experience poor health literacy or competing academic demands. Collectively, these findings indicate that interventions aimed at increasing PrEP utilization need to move beyond information provision towards strengthening risk appraisal, perceived benefits, self-efficacy and the practical capacity to incorporate PrEP into students’ routines.

## Theme 2

### Interpersonal level: interpersonal relationships and social influence on PrEP decision-making

The evidence demonstrates that PrEP decision-making is socially embedded and cannot be understood solely as an individual choice. Partners, peers, relationship dynamics, social expectations and gender norms influence how students interpret HIV risk and whether preventive practices are considered acceptable. Shamu et al. (23) identified the importance of supportive relationships in shaping preventive-health decisions among female university students, while Mthembu et al. (24) demonstrated that peer pressure, relationship-based trust, masculinity norms and transactional relationships contributed to sexual decision-making and inconsistent condom use (22, 23, 25–29). Tabata (29) similarly identified poor family communication and social influences among factors associated with risky sexual behaviors. Pillay et al. (27) further linked substance use and sexual decision-making to risky sexual behavior, with stronger associations observed among males.

Taken together, these findings indicate that students’ decisions regarding HIV prevention are negotiated within relationships rather than made in isolation. Interpersonal relationships may therefore operate in two opposing ways: they can provide social support and facilitate prevention, but they can also generate pressure, mistrust or expectations that discourage preventive practices. The importance of relationship trust is particularly significant because preventive behavior may be interpreted by partners as an indication of infidelity or perceived HIV risk. Similarly, gender expectations may influence whether students feel able to negotiate condoms or discuss PrEP openly. The evidence from the included studies suggests that these interpersonal dynamics may contribute to risky sexual behavior, while Shamu et al. (23) indicates that supportive relationships can facilitate engagement with prevention (29, 30).

The broader interpretation is that PrEP uptake should not be conceptualized as an exclusively individual act of health-seeking. A student may have adequate knowledge and positive intentions but still experience interpersonal constraints that affect disclosure, negotiation or continued use. The evidence therefore points towards the importance of interventions that recognize the social environment surrounding students, including peer influence, partner communication and supportive relationships. At the same time, the available evidence remains limited because relatively few studies directly examined interpersonal influences on PrEP utilization itself. Much of the evidence concerns HIV-risk behavior or contraceptive experiences, meaning that the relationship between interpersonal dynamics and actual PrEP use remains insufficiently explored.

## Theme 3

### Institutional level: accessibility, acceptability and responsiveness of PrEP services

The evidence suggests that institutional environments represent a critical point at which positive individual intentions can either be converted into PrEP utilization or become constrained. Across the included studies, barriers included waiting times, cost, inadequate information, provider attitudes, service location, clinic accessibility and interruptions in service delivery. Musakwa et al. (20) reported long waiting times, costs, academic scheduling conflicts, negative staff attitudes and inadequate health information as barriers to campus health-service utilization. Shamu et al. (26) similarly showed that young women valued convenience, discretion, supportive healthcare professionals and services that fitted their lifestyles, while Shamu et al. (23) identified health-system limitations, stigma and concerns about side effects as barriers to preventive-health uptake. The findings from Manyaka (18) also demonstrate that frequent clinic attendance and associated practical demands can reduce acceptability of CAB-LA despite its perceived effectiveness and privacy advantages (18).

The convergence of these findings suggests that availability of a PrEP service does not necessarily constitute meaningful accessibility. A service may technically exist within a university or surrounding community, yet remain difficult to use if students face long waiting periods, inconvenient operating arrangements, costs, unsupportive providers or concerns about confidentiality. This distinction is important because the evidence indicates that students’ utilization is shaped not only by whether PrEP is available but also by whether the service is perceived as acceptable, convenient and responsive to student circumstances.

The findings therefore reveal an implementation gap between service provision and service usability. Positive attitudes towards PrEP can be undermined when institutional systems impose practical or social costs on students. This may explain, in part, why high awareness and willingness reported in some studies do not consistently produce high uptake. The institutional environment consequently acts as a mediator between individual intention and actual preventive behavior. Strengthening campus-based services alone may therefore be insufficient unless those services are confidential, youth-friendly, affordable, convenient and supported by non-judgmental healthcare providers. However, the evidence base remains fragmented because several studies examined general HIV services rather than PrEP-specific institutional access.

## Theme 4

### Community/social level: stigma, misconceptions and social norms surrounding PrEP

The evidence indicates that the wider social environment shapes the meanings attached to PrEP and HIV prevention. Stigma, misconceptions, fear of judgement and prevailing social norms may influence whether students perceive PrEP as a legitimate preventive intervention or associate its use with negative social meanings. Haffajee et al. (22) identified misconceptions that PrEP could replace condoms, while Ntho et al. (9) found that nearly half of students believed PrEP could promote risky sexual behavior. Tabata (29) identified stigma, misconceptions and negative perceptions of health services in relation to risky sexual behavior and HIV prevention (29). Manyaka (18) demonstrated that privacy was one of the factors contributing to acceptance of CAB-LA, suggesting that discretion may be particularly important where prevention is socially sensitive (18). Shamu et al. (23) similarly identified stigma and negative social influences as barriers to uptake.

The findings suggest that PrEP is not interpreted solely according to its biomedical effectiveness. Its acceptability is also shaped by the social meanings attached to being a PrEP user. Misconceptions about risk compensation illustrate this tension: students may perceive PrEP as potentially encouraging unsafe sexual behavior rather than as an additional HIV-prevention strategy. Such beliefs can weaken motivation to initiate PrEP and may also influence attitudes towards people who use it. Stigma may therefore operate indirectly by increasing concerns about disclosure, judgement and social identity, even when students recognize the protective benefits of PrEP.

A key analytical finding is that community and social influences can either normalize or problematize prevention. Where PrEP is understood as a legitimate component of sexual-health prevention, social norms may support uptake; where it is associated with promiscuity, mistrust or increased sexual risk-taking, those same norms may discourage use. The evidence therefore suggests that improving PrEP utilization requires not only correcting individual misconceptions but also changing the broader social narratives surrounding PrEP. However, the available studies provide limited direct evidence on community-level influences among university students, with several findings inferred from stigma, social influence and HIV-risk behavior rather than directly examining community attitudes towards PrEP.

## Theme 5

### Structural level: socioeconomic and health-system conditions shaping PrEP utilization

The evidence demonstrates that structural conditions establish the broader boundaries within which students are able to access and sustain HIV-prevention practices. Socioeconomic circumstances, affordability, health-system capacity, service continuity and availability of information emerged across the included studies as factors that can constrain prevention. Musakwa et al. (20) identified costs, inadequate health information and limitations within university health services, while Manyaka (18) reported cost and access concerns in relation to CAB-LA. Shamu et al. (23) identified health-system limitations, while Pillay et al. (28) and Tabata (29) highlighted socioeconomic conditions as important influences on HIV-related risk behavior. Ntho et al. (9) additionally identified concerns about cost, accessibility, prescription requirements and rural access to youth-friendly HIV services.

The findings suggest that PrEP utilization is partly determined by conditions beyond the student’s individual control. A student may recognize personal HIV risk, perceive PrEP as beneficial and express an intention to use it, yet still be unable to translate that intention into practice when services are unaffordable, inaccessible or inconsistently available. This demonstrates why individual-level explanations alone are insufficient for understanding PrEP utilization. Structural conditions can effectively determine the range of choices that are realistically available to students.

The convergence between structural barriers and institutional barriers is particularly important. Institutional services are the immediate point of access, but their accessibility is influenced by broader health-system capacity, resource availability and socioeconomic inequalities. Consequently, improving PrEP uptake requires interventions that operate beyond student education, including affordable and continuous service provision, reliable medication availability, accessible delivery models and health systems capable of responding to the specific circumstances of young people. The evidence also suggests a need to consider geographical inequalities, particularly where students in rural or resource-constrained settings may experience fewer youth-friendly prevention options (9). Nevertheless, structural evidence in the included literature remains relatively underdeveloped, with several studies identifying socioeconomic conditions without directly examining how structural factors affect PrEP initiation or adherence.

## Theme 6

### HIV risk behaviors – patterns of sexual risk and their relationship to PrEP need

The included studies identified a range of HIV-related risk behaviors, including multiple sexual partnerships, inconsistent or condomless sex, alcohol- and drug-associated sexual behavior, and transactional or economically influenced sexual relationships (24, 27, 29, 30). Multiple sexual partnerships were reported across Limpopo, KwaZulu-Natal and the Western Cape, while inconsistent condom use was associated with relationship trust, peer influence, socioeconomic pressures and poor perceptions of freely available condoms (24, 27, 29, 30). Alcohol and drug use further appeared to contribute to risky sexual decision-making, including multiple partnerships and reduced condom use, with stronger associations reported among males (27). Analytically, these behaviors indicate potential HIV exposure and therefore provide an important context for PrEP eligibility and potential benefit; however, the presence of risk behavior did not consistently translate into recognition of HIV susceptibility or PrEP utilization. Thus, objective behavioral risk and students’ perceived need for PrEP may not always correspond.

The evidence further suggests that HIV risk is shaped by the context in which sexual behavior occurs rather than by individual behavior alone. Transactional relationships and socioeconomic challenges were associated with condomless sex, multiple partnerships and constrained prevention choices, while relationship trust, peer influence and social expectations also influenced condom use and sexual decision-making (24, 28, 29). These findings have important implications for PrEP decision-making because students experiencing situational or relationship-based risk may not consistently perceive themselves as being at risk, particularly when condomless sex occurs within a trusted relationship. At the same time, socioeconomic constraints may increase HIV exposure while limiting students’ ability to access, negotiate, disclose or consistently use prevention. The findings therefore suggest a potential mismatch between PrEP eligibility or potential benefit and perceived eligibility, whereby students may demonstrate behaviors that warrant consideration of PrEP but may not identify themselves as appropriate candidates.

A notable pattern across the evidence was the knowledge–behavior–perception gap, whereby some students demonstrated behaviors associated with HIV exposure despite inadequate HIV knowledge, misconceptions or a low perceived susceptibility to infection (6, 24, 27). This distinction is important because risky sexual behavior should not be interpreted as synonymous with PrEP uptake, nor should absence of PrEP use be interpreted as absence of HIV risk. Rather, the findings indicate that risk behavior may create the potential need for PrEP, while knowledge, perceived susceptibility, relationship circumstances, socioeconomic conditions and access to prevention services influence whether that potential need is recognized and acted upon. Consequently, PrEP interventions should address not only awareness of PrEP but also students’ understanding of their own HIV risk, particularly where risk is episodic, situational or normalized within relationships. Overall, the evidence maps a clear distinction between behavioral HIV risk and PrEP decision-making, while indicating that the relationship between the two is complex and requires further research directly examining how specific risk behaviors influence PrEP awareness, perceived eligibility, uptake, adherence and continued use.

## Discussion

The findings of this review indicate that PrEP awareness and willingness to use PrEP among South African undergraduate students do not consistently translate into actual uptake. This pattern is also evident in other sub-Saharan African settings, although the magnitude of the gap varies. For example, a study among university students in Nigeria reported PrEP awareness of only 18.9%, while only 1.5% of participants had ever used PrEP or PEP (31). More recent evidence from tertiary students in Calabar, Nigeria, demonstrates a somewhat different pattern, with 51.7% having heard of PrEP and 84.3% expressing willingness to use it, yet only 6% of sexually active participants reporting previous PrEP use (32). The comparatively higher awareness and willingness in the latter study may reflect increasing availability of PrEP information and greater exposure to HIV prevention programs. Nevertheless, the persistent gap between willingness and actual use resembles the pattern observed in South African students. Importantly, Nigerian students identified lack of information about where and how to obtain PrEP, limited availability, fear of side effects, friends’ disapproval, and perceived cost as barriers (32). These findings suggest that the transition from intention to utilization is influenced not only by individual knowledge but also by the availability, affordability, and social acceptability of PrEP services.

Evidence from other Low- and Middle- Income Countries (LMIC) settings further demonstrates that the relationship between awareness, willingness, uptake, and adherence is complex. Among young men who have sex with men who were students in China, 88.7% had heard of PrEP and 66.7% were willing to use it, but only 13.8% had taken PrEP and less than half of those who had used it reported adherence (33). This high-awareness/low-uptake pattern is particularly relevant to the present review because it demonstrates that increasing awareness alone may not overcome barriers occurring later in the PrEP continuum. In China, uptake was associated with residence in pilot cities and high-risk areas, suggesting that geographical availability and national implementation strategies can influence access to PrEP (33). Similarly, a comparison of YMSM students and non-students in China found high awareness but substantially lower use and adherence among both groups, with uptake associated with residence in pilot cities and high-risk areas (34). These findings suggest that differences in PrEP infrastructure and geographic availability may partly explain variations in uptake across settings. In South Africa, where PrEP has been incorporated into national HIV prevention efforts, the continued low uptake among some university students may therefore reflect barriers beyond simple availability, including stigma, perceived HIV risk, interpersonal influences, and the organization of student health services.

The South African findings can also be contrasted with evidence from countries where PrEP has been more established within health systems. Research among health-professions students in the United States found that 83.4% were aware of PrEP, although only 62.2% of final-year students reported having received PrEP education during their training (35). The higher awareness observed in this setting compared with some African student populations may partly reflect greater integration of PrEP into clinical education and a more mature PrEP program. However, important gaps remained even within this established program environment, particularly in how PrEP was presented to different populations; transgender people and heterosexual men and women were less frequently represented in PrEP education than men who have sex with men (35). This illustrates that established PrEP programs do not automatically eliminate knowledge or equity gaps. Rather, the content of health education, the populations targeted by prevention programs, and the extent to which PrEP is normalized across different groups can influence awareness and subsequent utilization.

Differences in health-system organization, health insurance, university infrastructure, and affordability may further explain variations between South Africa and other settings. In some LMIC contexts, direct and indirect costs, limited availability of PrEP, transportation difficulties, and inadequate healthcare infrastructure can restrict access even among individuals who are willing to use PrEP. For example, research among Chinese MSM identified accessibility and cost as important concerns influencing willingness to use PrEP (36), while Nigerian tertiary students similarly identified perceived expense and lack of availability as barriers (32). In contrast, where PrEP is more readily integrated into established healthcare systems, financial and geographical barriers may be reduced, shifting attention towards other factors such as stigma, side effects, perceived HIV risk, adherence, and acceptability. These contextual differences are important when interpreting the South African evidence because university students do not constitute a homogeneous population: students’ ability to access and sustain PrEP may vary according to socioeconomic circumstances, location, institutional resources, and access to confidential healthcare.

Cultural and interpersonal factors also provide an important explanation for differences across settings. In South Africa and other African contexts, PrEP decisions may occur within social environments where HIV-related stigma, concerns about sexuality, partner expectations, and gender norms influence preventive behavior. These influences may be particularly important for young women, who may experience difficulties negotiating prevention within relationships, while men and gender-diverse students may face different forms of stigma or may be less consistently reached by PrEP messaging. Evidence from Nigeria demonstrates that social disapproval and partner-related factors can influence PrEP awareness and use (31, 32), while evidence from China indicates that sexual behaviours, substance use, and residence in high-risk areas can influence progression through the PrEP cascade (33). Therefore, differences in uptake between countries should not be interpreted simply as differences in students’ knowledge or motivation. They may reflect differences in cultural norms, gender relations, perceptions of HIV risk, stigma, social networks, and the organization of health services.

### Gender dynamics, stigma and the campus health context

The findings of this review further indicate that gender should be understood as an analytical factor influencing PrEP decision-making rather than simply as a demographic characteristic. Gender relations may shape students’ perceptions of HIV vulnerability, health-seeking behavior, ability to negotiate prevention, and decisions regarding PrEP disclosure. This is particularly relevant for women, for whom HIV prevention decisions may occur within relationships characterized by unequal negotiating power. Evidence from South African studies among adolescent girls and young women indicates that concerns about partner reactions, relationship stability, and perceptions of infidelity can influence decisions regarding PrEP use and disclosure (37, 38). Although these studies are not exclusively focused on university students, they provide important contextual evidence for understanding how gendered relationship dynamics may affect young women’s prevention decisions.

Gender may also influence perceptions of vulnerability. Men may perceive themselves as less vulnerable to HIV or may be less likely to seek preventive healthcare because of socially constructed expectations surrounding masculinity and self-reliance. Conversely, women may experience pressure to demonstrate trust within relationships, making PrEP disclosure difficult where partners interpret PrEP use as evidence of infidelity or mistrust. Evidence from South Africa demonstrates that partner reactions and relationship concerns can influence PrEP acceptability and uptake among young women (37, 38). These findings indicate that PrEP decisions cannot always be interpreted as entirely autonomous individual choices because gender power relations may shape the extent to which students are able to negotiate HIV prevention.

Stigma also emerged as an important factor requiring consideration beyond HIV-related stigma alone. PrEP-related stigma may occur even where HIV stigma is not explicitly present. Students may fear that taking PrEP will lead peers, partners, family members, or healthcare providers to assume that they are engaging in risky sexual behavior, have multiple partners, or are promiscuous. Consequently, a student may avoid or conceal PrEP use because of anticipated social judgement rather than because of concerns about HIV itself (10, 38). PrEP-related stigma can therefore arise from the visibility of prevention behavior, particularly when daily pill-taking raises questions from partners or peers.

The evidence also suggests that stigma operates across several levels. HIV-related stigma may create fear of being associated with HIV; PrEP-related stigma may arise from being identified as a PrEP user; sexual-behavior stigma may result from assumptions about promiscuity or multiple partnerships; partner stigma may involve fear of judgement or mistrust; and provider stigma may occur when students anticipate judgement during healthcare encounters. Qualitative evidence among female college students in Durban identified fear of judgement, misconceptions, concerns about side effects, and partner-related concerns as barriers to PrEP demand (10). Evidence among young people in South Africa also indicates that healthcare-provider interactions and service environments can influence PrEP experiences (11). Thus, stigma should be understood as a social and health-service barrier, rather than solely as an individual perception.

This distinction has important implications for interpreting the low uptake identified in this review. If students avoid PrEP because they fear being perceived as promiscuous, increasing knowledge about PrEP alone may not resolve the problem. Similarly, if students fear judgement from healthcare providers, simply making PrEP available within a campus clinic may not result in increased utilization. Interventions therefore need to address stigma at the individual, interpersonal, community, and institutional levels. Nevertheless, the available evidence does not permit the extent of HIV-related stigma, PrEP-specific stigma, sexual-behavior stigma, and provider stigma to be quantified specifically among South African undergraduate students. Further qualitative and mixed-methods research is required to examine how these forms of stigma interact and whether their effects differ according to gender, relationship status, socioeconomic circumstances, and institutional context.

The findings should also be interpreted within the campus health-service environment. Universities and colleges provide an important opportunity for HIV prevention because students can potentially access PrEP, HIV testing, counselling, and related services within or near their educational institutions. However, the presence of a campus health facility does not necessarily mean that PrEP is readily accessible. Factors such as whether clinics provide PrEP, availability of HIV testing and counselling, medication supply, operating hours, waiting times, confidentiality, provider attitudes, and the extent to which services are perceived as student-friendly may influence whether students’ progress from awareness and willingness to actual PrEP utilization.

South African evidence among young people identifies structural barriers including service accessibility, waiting times, confidentiality concerns, inadequate information, and difficulties interacting with healthcare providers (11, 39). These factors may be particularly relevant to undergraduate students who have academic commitments and may be reluctant to attend clinics during teaching hours or make repeated visits for PrEP follow-up. Confidentiality may also be particularly important in university environments where students and healthcare personnel may know one another, increasing concerns about unwanted disclosure of sexual-health information.

Provider attitudes and competencies represent another important component of the implementation environment. A student may be willing to use PrEP but decide not to initiate it if the consultation is perceived as judgmental, if insufficient information is provided, or if concerns about side effects and adherence are not adequately addressed. Conversely, supportive and well-trained providers may facilitate informed decision-making and continuation. Evidence from PrEP implementation among young women in South Africa highlights the importance of healthcare-provider engagement, communication, training, and supportive service environments (37, 40). Provider training should therefore extend beyond technical knowledge of PrEP to include non-judgmental communication, confidentiality, gender-sensitive care, sexuality-affirming approaches, and stigma reduction.

The availability of PrEP within campus health services is consequently only one component of effective implementation. Student-friendly services should ideally integrate PrEP with HIV testing, counselling, adherence support, sexual and reproductive health services, and appropriate follow-up, while ensuring reliable medication availability and convenient access. Where campus clinics do not provide PrEP directly, clear referral pathways to accessible external services may be necessary. However, the evidence identified in this review is insufficient to determine systematically how consistently South African universities and colleges currently provide these services. In particular, evidence comparing urban and rural institutions, historically underserved universities, and TVET colleges remains limited.

### Evidence gaps

The available South African evidence on PrEP among students in higher education remains limited and unevenly distributed across institutions and student groups. Existing research has predominantly focused on university students, particularly those in selected urban or well-established institutions, while evidence from rural universities, historically underserved institutions, and technical and vocational education and training (TVET) colleges remains limited. Recent research from six universities in Gauteng demonstrates continued interest in factors influencing PrEP uptake, but the geographical concentration of such studies limits understanding of PrEP use across the broader South African higher-education sector (27). Evidence is also limited regarding differences in PrEP awareness, acceptability, uptake, and adherence across students from different socioeconomic backgrounds. Furthermore, although research has examined PrEP among young women and other populations in South Africa, evidence specifically addressing male students and gender-diverse students within higher education settings remains limited (37, 41).

Important gaps are also evident across the PrEP continuum. Existing student-focused research has provided greater attention to awareness, knowledge, attitudes, willingness, and initial uptake than to sustained PrEP use, particularly adherence, persistence, and discontinuation (6, 27). For example, research among university students in KwaZulu-Natal identified substantial gaps in comprehensive PrEP knowledge, despite students having some awareness of PrEP (22). More recent evidence among South African health science students similarly demonstrates high awareness and behavioral intention but identifies gaps in specific PrEP knowledge (9). Although long-acting injectable PrEP is increasingly being investigated in South Africa, evidence specifically examining its acceptability, uptake, persistence, and implementation among undergraduate students remains limited. Current South African research on newer PrEP modalities has largely focused on young women or broader adolescent and young adult populations rather than higher-education students specifically (26). There is also limited evidence examining implementation processes, healthcare-provider perspectives, institutional policies, and delivery models for PrEP within universities and colleges.

Further gaps concern the structural and service-delivery dimensions of PrEP use. Evidence specifically examining cost and affordability, accessibility of PrEP services, institutional policies, digital interventions, and mechanisms for supporting PrEP persistence among South African undergraduate students remains limited. While implementation research among other young populations in South Africa has highlighted the importance of healthcare providers, peer support, stigma, communication, and service-delivery environments, these findings cannot automatically be generalized to students in higher education because of differences in population and context (37, 42). Similarly, qualitative evidence provides valuable insights into students’ perceptions, barriers, and facilitators, but remains less extensive than quantitative evidence in the South African university context (10). Therefore, the principal gap is not necessarily a complete absence of evidence, but rather the limited breadth, uneven geographical and institutional coverage, and concentration of research on selected stages of the PrEP continuum. These gaps support the need for this scoping review to systematically map the existing evidence and identify under-researched populations, settings, PrEP outcomes, and implementation issues within South African higher education.

The available evidence is geographically concentrated, particularly in KwaZulu-Natal and Gauteng, with comparatively limited evidence from other provinces and from rural, historically underserved, and resource-constrained higher-education institutions. The evidence is also predominantly based on cross-sectional quantitative studies, limiting understanding of changes in PrEP use over time.

### Future research recommendations

Based on the findings of this scoping review, several recommendations are proposed to improve PrEP uptake and reduce HIV-related risk behaviors among undergraduate students in South African higher education institutions. Firstly, higher education institutions, in collaboration with the Department of Health and campus health services, should strengthen comprehensive and continuous HIV prevention education, with specific emphasis on improving accurate knowledge of PrEP and distinguishing it from PEP. Secondly, university management and student wellness centers should expand accessible, youth-friendly, and stigma-free PrEP services on campus, ensuring confidentiality, reduced waiting times, flexible service hours, and reliable access to PrEP to accommodate students’ academic schedules. Thirdly, healthcare providers working in campus and community clinics should receive targeted training to improve their knowledge, attitudes, communication, and counselling practices, particularly in relation to PrEP eligibility, adherence, confidentiality, and stigma, thereby improving students’ willingness to initiate and continue PrEP. In addition, policymakers and the Department of Health should support the integration of innovative HIV prevention strategies, including long-acting injectable PrEP, into existing student health programs, accompanied by appropriate education to address concerns, misconceptions, and potential stigma surrounding new PrEP modalities.

Future research should address the specific evidence gaps identified in this review. Longitudinal studies are needed to examine the transition from PrEP awareness and willingness to initiation, as well as patterns of adherence, persistence, and discontinuation among undergraduate students. Qualitative studies should explore students’ lived experiences and decision-making regarding PrEP, including the influence of perceived HIV risk, stigma, partner and peer relationships, gender norms, and concerns about disclosure. Implementation research should assess how PrEP can be effectively delivered through campus health services, including the availability of trained providers, confidentiality, accessibility, medication supply, service hours, referral pathways, and integration with HIV testing and counselling. Research should also examine provider knowledge, attitudes, and practices regarding PrEP and determine how provider-related factors influence student uptake and continuation. Given the increasing availability of alternative PrEP modalities, studies should specifically evaluate the acceptability, uptake, persistence, and implementation of long-acting injectable PrEP among university and college students in South Africa. Further research should examine how gender and relationship dynamics influence PrEP decision-making, disclosure, negotiation of HIV prevention, and continued use, while ensuring that evidence gaps concerning male and gender-diverse students are explicitly addressed.

Finally, intervention studies are needed to determine whether campus-based PrEP education and service-delivery interventions can improve PrEP initiation, adherence, and persistence. Such studies should evaluate the effectiveness of digital interventions, including mobile health platforms, social media-based education, digital counselling, and appointment or adherence-support systems, as well as peer-led interventions that use trained students to provide PrEP information, support, and referrals. Comparative studies should determine which approaches are most acceptable, feasible, and effective across different South African higher-education settings, including urban, rural, and historically underserved institutions. Collectively, these studies would move the evidence base beyond describing barriers towards identifying effective and contextually appropriate strategies for improving sustained PrEP use among students in South African higher education institutions.

### Policy and practice implications

The findings of this scoping review have important implications for universities, healthcare providers, and policymakers responsible for HIV prevention among students in South African higher education institutions. Universities and colleges should integrate PrEP education into student orientation programs and incorporate PrEP information into existing HIV prevention, sexual and reproductive health, and student wellness programs. This should include clear and accurate information on PrEP, its effectiveness, eligibility, adherence, and the distinction between PrEP and PEP. Campus health services should be strengthened to provide confidential, accessible, youth-friendly, and student-centered PrEP services, including HIV testing, counselling, PrEP initiation, follow-up, and adherence support. Flexible clinic hours, reduced waiting times, and reliable PrEP availability should be considered to accommodate students’ academic schedules. Where PrEP is not available on campus, institutions should establish effective referral mechanisms with nearby community health facilities to ensure continuity of care.

Healthcare providers should receive targeted training on PrEP provision, communication, confidentiality, and non-judgmental and stigma-sensitive care. Universities should also develop peer educator programs in which trained students provide PrEP information, challenge misconceptions, encourage appropriate service utilization, and facilitate referrals to campus health services. Digital platforms, including student portals, university websites, and social media, should be used to provide accurate and accessible PrEP information and communicate where and how students can obtain services. Policy and practice should explicitly address PrEP-related stigma, including perceptions that PrEP use indicates promiscuity, infidelity, or HIV infection. PrEP should be integrated into routine HIV testing and sexual and reproductive health services rather than delivered as an isolated intervention. Furthermore, stronger linkages between campus and community health services are needed to support students who are away from campus, change institutions, or require services beyond the capacity of campus clinics. Collectively, these measures could create a more supportive and accessible environment for PrEP uptake and continued use among undergraduate students.

### Study strengths and limitations

This manuscript has several notable strengths. First, it addresses an important public health issue, as PrEP represents a critical component of HIV prevention in South Africa. Second, the focus on university and college students addresses an important and potentially underexplored population within HIV prevention research. The use of a scoping review methodology is appropriate for systematically mapping heterogeneous evidence across qualitative, quantitative, and mixed-methods studies. The application of established methodological guidance, including the Joanna Briggs Institute (JBI) guidance and the Preferred Reporting Items for Systematic Reviews and Meta-Analyses extension for Scoping Reviews (PRISMA-ScR), further strengthens the methodological foundation of the review.

The inclusion of gray literature potentially reduces the risk of overlooking relevant unpublished and implementation-related evidence, while the thematic synthesis enables the review to move beyond individual study findings and identify broader patterns across the evidence base. Furthermore, the findings have potential policy and practice relevance for university health services, HIV prevention programs, and national PrEP implementation strategies. Overall, by providing a focused synthesis of evidence on PrEP awareness, acceptability, uptake, adherence, and HIV-related risk behaviors among university students in South Africa, this review addresses an important evidence gap and may contribute to strengthening HIV prevention within higher education settings.

This scoping review also has limitations. The review included a relatively small number of studies, with a predominance of quantitative and cross-sectional designs and limited longitudinal and qualitative evidence, which restricts understanding of changes in PrEP utilization over time and students’ lived experiences. The search was restricted to English-language publications and selected electronic databases and grey-literature sources, which may have resulted in language and database coverage bias, while publication bias cannot be completely excluded despite backward and forward citation tracking. The evidence base was also heterogeneous, with variations in the definitions and measurement of PrEP awareness, acceptability, uptake, adherence, and HIV-related risk behaviors, making direct comparison across studies challenging. Geographical concentration of studies may have resulted in underrepresentation of rural universities, historically underserved institutions, and other higher-education settings. Differences in methodological quality across included studies may also influence interpretation of the findings. In addition, the included studies were conducted across different periods of PrEP availability, national implementation policies, and service-delivery environments in South Africa; consequently, changes in the policy environment and access to PrEP over time may have influenced reported findings. Finally, because this was a scoping review, the synthesis was intended to map the available evidence on PrEP awareness, acceptability, uptake, adherence, and HIV-related risk behaviors among university students in South Africa, rather than establish causal relationships between these concepts.

## Conclusion

This scoping review mapped the evidence on PrEP awareness, acceptability, uptake, adherence, and HIV-related risk behaviors among university students in South Africa and identified six interrelated evidence domains: individual perceptions, knowledge and appraisal of HIV and PrEP; interpersonal relationships and social influences on PrEP decision-making; accessibility, acceptability and responsiveness of PrEP services; stigma, misconceptions and social norms surrounding PrEP; socioeconomic and health-system conditions; and HIV-related risk behaviors and their relationship with PrEP. Collectively, the evidence suggests that although awareness and knowledge of PrEP are important components of HIV prevention decision-making, they do not necessarily correspond with PrEP acceptability, uptake or adherence.

These outcomes appear to be situated within interacting individual, interpersonal, institutional and structural contexts, while HIV-related risk behaviours provide an important context for understanding students’ engagement with PrEP. The findings therefore indicate that improving PrEP awareness alone is unlikely to address the range of factors reflected across the evidence. Efforts to strengthen PrEP engagement among university students should adopt a multidimensional approach that addresses informational needs alongside stigma, social influences, service accessibility and responsiveness, socioeconomic and health-system barriers, and the diverse HIV-related risk contexts experienced by students. Further research is needed to strengthen the evidence base across these domains and to examine how PrEP awareness, acceptability, uptake and adherence relate to HIV-related risk behaviours among diverse student populations and university settings in South Africa.
