# Supplementary material for: A scoping review of pre-exposure prophylaxis awareness, acceptability, uptake, adherence, and HIV-related risk behaviors among university students in South Africa
Source: Front Public Health. 2026 Oct 15;14:1907622. doi: 10.3389/fpubh.2026.1907622 (PMC13717243; doi:10.3389/fpubh.2026.1907622)
Supplement: Supplementary file 1 [file Data_Sheet_1.pdf]

Supplementary 1: Title and Abstract Screening Form

No.	Screening question	Yes	No	Unclear
1	Is the study conducted in South Africa?			
2	Does the study involve undergraduate students?			
3	Is the study conducted in a university, university of technology, college, or TVET college?			
4	Does the study address PrEP awareness, knowledge, acceptability, willingness, uptake, initiation, utilization, adherence, persistence, or discontinuation?			
5	Does the study address HIV-			

	related risk behaviours relevant to the review?			
6	Was the study published between 2014 and 31 May 2026?			
7	Is the publication available in English?			
8	Does the study appear to meet the eligibility criteria based on the title and abstract?			

Screening decision:

☐ Include

☐ Exclude

☐ Maybe/Unclear – retrieve full text

Reason for exclusion:

____

Supplementary 2: Data Extraction Tool

Section	Data to extract
Study identification	Author(s), year of publication, study title
Study characteristics	Country, study setting, study design/methodology
Participant characteristics	Sample size, age range/mean age, sex/gender, population/key population
PrEP-related information	PrEP awareness, knowledge, acceptability, uptake, adherence, persistence/discontinuation
HIV-related risk behaviours	Multiple sexual partners, condom use/non-use, transactional sex, substance/alcohol use, STIs, sexual partner characteristics, other reported risk behaviours
Determinants/factors	Individual factors, interpersonal/social factors, institutional/service-delivery factors, structural factors, product-related factors
Socio-ecological level	Individual; interpersonal/social; institutional/service-delivery; structural; product-related
Key findings	Main findings relevant to the research questions
Barriers	Reported barriers to PrEP awareness, access, uptake or adherence
Facilitators	Factors facilitating PrEP use/adherence
